# Enhancing short-packet communications: BLER performance in RIS-assisted ambient backscatter NOMA systems

**DOI:** 10.1371/journal.pone.0328545

**Published:** 2025-08-05

**Authors:** Le Si Phu, Tan N. Nguyen, Bui Vu Minh, Phu Tran Tin, Miroslav Voznak

**Affiliations:** 1 Faculty of Electrical Engineering and Computer Science, VSB-Technical University of Ostrava, Ostrava, Czechia; 2 Advanced Intelligent Technology Research Group, Faculty of Electrical and Electronics Engineering, Ton Duc Thang University, Ho Chi Minh City, Vietnam; 3 Faculty of Engineering and Technology, Nguyen Tat Thanh University, Ho Chi Minh City, Vietnam; 4 Data Science Laboratory, Faculty of Information Technology, Ton Duc Thang University, Ho Chi Minh City, Vietnam; University of Jeddah, SAUDI ARABIA

## Abstract

Short-packet communication (SPC) is a key enabler for ultra-reliable low-latency communication (URLLC) in next-generation wireless networks. In this paper, we investigate the block error rate (BLER) performance of a reconfigurable intelligent surface (RIS)-assisted ambient backscatter communication (AmBC) system operating under a non-orthogonal multiple access (NOMA) framework. Different from existing works, our study considers a Nakagami-*m* fading environment and derives closed-form expressions for the average and asymptotic BLER. By leveraging power-domain NOMA and RIS-assisted reflection, we optimize signal reception for both direct and backscattered links, enhancing spectral efficiency and communication reliability. Numerical and Monte Carlo simulation results validate our analytical findings, demonstrating significant performance gains in terms of BLER reduction and system throughput improvement. These results highlight the potential of RIS-assisted AmBC-NOMA systems in enabling efficient SPC for URLLC applications.

## 1 Introduction

Internet of Things (IoT) has become a pivotal technology for next-generation wireless communication systems, enabling pervasive connection for many sensor nodes and IoT devices over the internet [1–4]. In addition to the requirements for high throughput and enhanced spectral efficiency, the sixth generation (6G) necessitates that wireless infrastructure incorporate advanced ultra-reliability and low-latency communication (URLLC) capabilities, achieving a communication reliability of 99.99% and maintaining transmission latency within one millisecond [5–7]. Consequently, in light of more rigorous and multiuser communication demands, there is an urgent need for more sophisticated technologies that can enhance transmission reliability and facilitate extensive connections.

Recently, the idea of reconfigurable intelligent surfaces (RISs) has been extensively examined owing to its capacity to convert the uncertain wireless channel into an omnipresent network [8–10]. RIS is a configuration of several reflecting elements (REs) that may independently modify the reflected signal with the desired phase shift. Consequently, by appropriately adjusting the phase, RIS creates an advantageous channel response, hence providing an extra degree of flexibility to enhance capacity and extend coverage. Moreover, in addition to enhancing the received signal strength at the intended destination, RIS may also direct the reflected beam away from an eavesdropper, so ensuring secure communication, and RIS is a cost-effective and energy-efficient smart radio environment technology that lacks power-consuming components and enhances performance with little power use [11, 12]. Besides, ambient backscatter communication (AmBC), with passive radio frequency (RF) identification capabilities, have emerged as a viable paradigm for the deployment of IoT systems [13, 14]. A reader sends a segment of RF signals for reflection and modulation to convey information, while the remaining piece is transformed into energy for circuit operation. Furthermore, when integrated with RIS, it generates varied signal pathways and a resilient, energy-efficient communication framework [15–17]. Specifically, the authors in [15] investigated the performance of RIS on discrete connections inside AmBC by computing the average bit error rate between RIS and AmBC links. In [16], the authors evaluated the performance of a RIS-assisted AmBC system, deriving closed-form expressions for outage probability (OP) and average symbol error rate (ASER), while also providing asymptotic OP and diversity order to enhance understanding. The work in [17] analyzed secrecy performance by establishing closed-form and asymptotic equations for secrecy outage probability in two scenarios: imperfect successive interference cancellation (ipSIC) and perfect SIC (pSIC).

Furthermore, non-orthogonal multiple access (NOMA) is recognized as an efficient multiple-access method to address the substantial connection requirements of IoT networks. In power-domain NOMA, several users are accommodated on the same resource block by multiplexing their data in the power domain, in contrast to the orthogonal resource allocation (e.g., frequency, time, and code) used by traditional orthogonal multiple access (OMA) methods [18–20]. Numerous studies on NOMA have explored their contributions across various application scenarios under different assumptions, including RIS [21–25] and backscatter communication [26–29]. In the RIS-assisted NOMA systems, the authors in [26] presented performance measures such as secrecy outage probability (SOP) and average secrecy capacity (ASC) in a closed-form approximation in the context of physical layer security. While, the authors in [22] expressed the closed-form equations for the OP, throughput, and an upper limit for the ergodic capacity (EC) under multi-primary user limitations, considering two scenarios of line-of-sight (LoS) link from the source to users. Furthermore, the authors in [23] have proposed the performance of RIS-assisted NOMA vehicular networks, considering the effects of ipSIC by deriving closed-form expressions for pairwise error probability (PEP) to evaluate the union bound on the bit error rate of NOMA users. The closed-form expressions OP, SE, and EE are derived in [24] with the hardware impairments, and imperfect SIC in the downlink RIS-assisted NOMA wireless networks in the context of phase error at RIS. In the cooperative-NOMA system utilizing multiple-user RIS-assisted AmBC, the authors studied performance by employing an unmodulated carrier, Binary Phase-shift Keying (BPSK), Band Pass Filter (BPF), and Successive Interference Cancellation (SIC) for signal decoding, deriving exact closed-form expressions for the OP [26]. The authors in [27], have introduced the zero-energy RIS (Ze-RIS) supported AmBC system utilizing NOMA, wherein several critical parameters, including the amplitude reflection coefficient, reflection coefficients, transmit beamforming, and passive beamforming, are optimized to enhance the system’s energy efficiency. In [28], the optimization issue of the beamforming vector is formulated to optimize the weighted sum rate of the secondary users while maintaining quality of service (QoS) for the primary user in the active RIS AmBC-enabled NOMA network inside a cognitive radio system. The authors in [29] have introduced the RIS-enhanced NOMA-AmBC system, deriving the closed-form formula for OP under ipSIC and characterizing the channel according to Nakagami-m fading.

Conversely, many application situations in IoT need the sharing of little information across nodes. Consequently, short packet communication (SPC) is progressively emerging as a characteristic of the IoT. The Shannon capacity theory is inapplicable in this scenario, because transmission reliability cannot be assured at excessively high signal-to-noise ratios (SNRs) [30–32]. The authors in [33] have studied the performance of RIS-assisted NOMA in SPC under imperfect SIC by establishing a closed-form formula for the average block error rate (BLER) with both random and optimum phase shifts at RIS. Meanwhile, [34] introduced a multi-antenna RIS-assisted SPC, deriving closed-form formulas for BLER, throughput, latency, and reliability to assess the suggested systems by combining the technique maximum-ratio transmission beamforming, and selection combining or maximum-ratio combining. The authors in [35] investigated RIS-assisted NOMA with cognitive radio inside the IoT framework by articulating the average BLER for primary and secondary users in closed-form expressions. The study in [36] introduced a closed-form and asymptotic expression for average BLER to evaluate the performance of RIS-assisted NOMA in SPC, including the effects of hardware impairments. In [37], the authors addressed the optimization problem of maximizing the sum rate and sensing power through the optimization of the transmit beamformer, phase shifts, quality of service constraints, and unit modulus phase shift, utilizing the alternate optimization and successive convex approximation methods.

Based on the benefits mentioned above, in the present paper, we propose the performance of the RIS-assisted AmBC system under the Nakagami-*m* distribution. Table 1 offers a concise comparison between our proposed scheme and representative state-of-the-art studies in the literature. The main contributions and novelties of the present work are summarized as follows:

- We derive closed-form expressions for the average and asymptotic BLER in a RIS-assisted AmBC system operating under a NOMA framework. Unlike existing works, our analysis is conducted under a Nakagami-*m* fading environment, capturing practical wireless channel conditions more accurately.
- We analyze the effect of the number of reflecting elements *N* in RIS on system reliability. Our findings reveal that increasing *N* significantly enhances the performance of the far user, enabling RIS to compensate for severe path loss and improve signal reception in AmBC-NOMA systems.
- We compare the BLER performance of NOMA and OMA in RIS-assisted AmBC systems, demonstrating that NOMA consistently outperforms OMA, especially in low-SNR and high-fading scenarios. This highlights the superiority of NOMA in resource allocation and spectral efficiency for SPC.
- We evaluate the impact of blocklength L on BLER and system reliability, uncovering the trade-offs between latency and error performance. Our results show that while increasing L improves reliability, the gains diminish at high values of L, emphasizing the need for optimized blocklength selection in URLLC scenarios.

**Table 1:** Comparison of the proposed system with existing works.

Technologies	Our Scheme	[16]	[38]	[39]	[40]	[41]
NOMA	✓	X	✓	✓	✓	✓
RIS	✓	✓	X	X	X	✓
AmBC	✓	✓	X	X	X	✓
Imperfect SIC	✓	X	X	X	X	X
Nakagami-*m* fading	✓	✓	✓	X	X	✓
Average BLER	✓	X	X	✓	✓	✓
Asymptotic	✓	✓	✓	X	✓	X
Reliability	✓	X	X	X	X	X
Throughput	✓	X	✓	✓	X	✓
Optimization	X	X	✓	✓	X	X

The remainder of this paper is organized as follows. Sect 2 introduces the proposed system model and provides an analysis of the transmitted signals. In Sect 3, we present the channel characteristics, an overview of the blocklength error rate, as well as the closed-form expressions for the average BLER and its asymptotic behavior. Sect 4 discusses the numerical results, while Sect 5 concludes the paper.

The main notations of this paper is shown as follows: $CN(\mu ,\sigma^{2})$: complex Gaussian distribution; $f_{X}(\cdot ),F_{X}(\cdot )$: PDF and CDF; $I_{v}(\cdot )$: modified Bessel function of the first kind; γ(a,x): lower incomplete gamma function; Γ(·): Gamma function; $G_{p,q}^{m,n}\left(x|\begin{array}{c} a_{1},\ldots ,a_{p}\\ b_{1},\ldots ,b_{q} \end{array}\right)=\frac{1}{2\pi i}\int \frac{\prod_{j=1}^{m}\Gamma \left(b_{j}-s\right)\prod_{j=1}^{n}\Gamma \left(1-a_{j}+s\right)}{\prod_{j=m+1}^{q}\Gamma \left(1-b_{j}+s\right)\prod_{j=n+1}^{p}\Gamma \left(a_{j}-s\right)}x^{s}ds$: Meijer G-function [42, Eq (9.301)]; ${\;}_{2}F_{1}\left(a,b;c;z\right)=\sum_{k=0}^{\infty }\frac{{\left(a\right)}_{k}{\left(b\right)}_{k}z^{k}}{{\left(c\right)}_{k}k!}$: Gauss hypergeometric function [42, Eq (9.100)]; ${\left(x\right)}_{k}$: Pochhammer symbol; $Q(x)=\frac{1}{\sqrt{2\pi }}\int_{x}^{\infty }e^{-t^{2}/2}\;dt$: Gaussian Q-function.

## 2 System model

**Fig 1:**
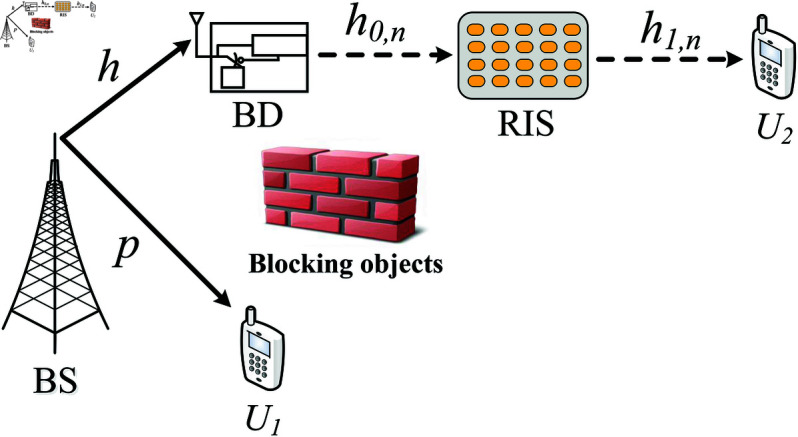
Illustration of a RIS-assisted AmBC with NOMA system model.

We consider a RIS-assisted AmBC system, as illustrated in Fig 1. The system consists of a base station (BS), a RIS with *N* reflecting elements, a backscatter device (BD), and two users: a near user (*U*_1_) and a far user (*U*_2_). The network operates based on NOMA to enhance spectrum efficiency and support multiple users. We assume BS, BD, *U*_1_, and *U*_2_ are each equipped with a single antenna. The BS transmits continuous sinusoidal carrier signals, which serve as both an energy source for the BD and a communication signal for the users. The BD employs ambient backscatter modulation by dynamically adjusting its load impedance, thereby embedding its own information into the incident carrier wave. The direct communication between the BS and *U*_1_ occurs without the assistance of either the BD or the RIS. However, to extend the coverage and enhance signal quality for both BD and *U*_2_, the RIS is strategically deployed to reflect and enhance the signals. Specifically, the RIS not only assists in forwarding BD’s backscattered signal but also strengthens the reception at *U*_2_, compensating for severe path loss over long distances. Due to environmental obstructions, we assume that the direct links from the BS to the RIS, BD, and *U*_2_ are blocked. As a result, the RIS plays a crucial role in facilitating communication by intelligently reconfiguring its phase shifts to optimize the received signals at both BD and *U*_2_. Meanwhile, NOMA is applied to allocate power efficiently between *U*_1_ and *U*_2_, ensuring that users with different channel conditions can effectively decode their respective signals. This RIS-assisted AmBC-NOMA system has the potential to significantly improve spectral and energy efficiency, making it a promising candidate for next-generation wireless networks. An enumeration of the critical symbols is provided in Table 2.

**Table 2:** List of key symbols.

Symbol	Description
*N*	Number of RIS reflecting elements
BS	Base station
BD	Ambient backscatter device
$U_{1},\;U_{2}$	Near user and far user, respectively
*h*	Channel coefficient BS → BD
$h_{0,n},\;h_{1,n}$	Channel coefficients BD → RIS and RIS → U_2_
*p*	Direct channel coefficient BS → U_1_
*P*_*S*_	Transmit power at the base station
$a_{1},\;a_{2}$	Power allocation coefficients for *x*_1_ and *x*_2_
L	Blocklength
$B_{1},\;B_{2}$	Number of information bits of *x*_1_ and *x*_2_
ζ	SIC imperfection factor (0 for perfect SIC)
$m,\;m_{g_{0}},\;m_{g_{1}},\;m_{p}$	Nakagami–*m* fading shape parameters
$\Omega_{g_{0}},\;\Omega_{g_{1}},\;\Omega_{p}$	Average channel power gains
$\rho_{S}$	Average transmit SNR ($P_{S}/\sigma^{2}$)
$\gamma_{U_{2},x_{2}},\;\gamma_{U_{1},x_{i}}$	Instantaneous SINRs for decoding *x*_2_ at U_2_ and *x*_*i*_ at U_1_
*r*_*i*_	Coding rate *B*_*i*_/*L* for user *i*
${\bar{\epsilon }}_{i}$	Average BLER of user *i*
$\tau_{i},\;\tau_{\text{sys}}$	Throughput and system throughput

### 2.1 Analysis of signals at downlink

Denoting $h\in {\mathbb{C}}^{1\times 1}$ as the channel coefficient from BS to BD, ${\vec{h}}_{0}\in {\mathbb{C}}^{N\times 1}$ and ${\vec{h}}_{1}\in {\mathbb{C}}^{N\times 1}$ as the channel vectors from BD-RIS and RIS to *U*_2_, respectively. Additionally, we denote $p\in {\mathbb{C}}^{1\times 1}$ as the direct channel from BS to *U*_1_. In this work, we consider a general Nakagami-*m* fading distribution for all transmission links. Moreover, all channel coefficients associated with the RIS are assumed to be independent and identically distributed (i.i.d.) and RIS controller can acquire perfect channel state information (CSI). To enhance spectrum efficiency, power-domain NOMA is adopted at the BS. The transmitted signal at BS is given by:

$$x_{S}=\sqrt{a_{1}P_{S}}x_{1}+\sqrt{a_{2}P_{S}}x_{2},$$ (1)

where *x*_1_ and *x*_2_ represent the signals intended for *U*_1_ and *U*_2_, respectively. The power allocation coefficients *a*_1_ and *a*_2_ satisfy $a_{1}+a_{2}=1$ with $0<a_{1}<a_{2}<1$ to ensure that the far user *U*_2_ receives a stronger power level. The transmitted signals are normalized such that $𝔼\left\{{\left|x_{1}\right|}^{2}\right\}=𝔼\left\{{\left|x_{2}\right|}^{2}\right\}=1$ and *P*_*S*_ is the power transmit power of BS. The received signal at *U*_1_ with the direct link from BS is given by

$$y_{U_{1}}=px_{S}+\omega_{U_{1}}=p\left(\sqrt{a_{1}P_{S}}x_{1}+\sqrt{a_{2}P_{S}}x_{2}\right)+\omega_{U_{1}}.$$ (2)

Meanwhile, *U*_2_ relies on BD and RIS to receive the signal as

$$y_{U_{2}}=h_{1}^{T}\Phi h_{0}hx_{S}+\omega_{U_{1}}\;=h_{1}^{T}\Phi h_{0}h\left(\sqrt{a_{1}P_{S}}x_{1}+\sqrt{a_{2}P_{S}}x_{2}\right)+\omega_{U_{2}},$$ (3)

where $\Phi =\text{diag}(\alpha_{1}e^{j\theta_{1}},\ldots ,\alpha_{n}e^{j\theta_{2}},\ldots ,\alpha_{N}e^{j\theta_{N}})$ represents a diagonal matrix, with $0<\alpha_{n}\leq 1$, n∈{1,…,N} indicating the amplitude reflection coefficient for the *n*-th reflecting element, and $\theta_{n}\in [0,2\pi )$ denoting the phase shift of the *n*-th reflecting element. Here, $e^{\left(.\right)}=\exp(.)$ is the exponential function, ${\vec{h}}_{1}=[h_{1,1},\ldots ,h_{1,n},\ldots ,h_{1,N}{]}^{T}$, ${\vec{h}}_{0}=[h_{0,1},\ldots ,h_{0,n},\ldots ,h_{0,N}{]}^{T}$, where $(.){\;}^{\text{T}}$ indicates the transpose operation. Let $h=ge^{j\vartheta }$, $h_{1,n}=g_{1,n}e^{j\phi_{n}}$, and $h_{0,n}=g_{0,n}e^{j\varphi_{n}}$ signify the channel coefficients from BS to BD, and from BD to the *n*-th element of the RIS. The channel from BS to BD and from BD to RIS creates a cascade channel from BS to RIS due to backscatter modulation, and then from the *n*-th RIS element to *U*_2_, respectively. $\vartheta ,\phi_{n},\varphi_{n}\in [0,2\pi )$ are the phase shifts related to *h*, $h_{1,n},\;\text{and}\;h_{0,n}$. g=|h|, $g_{1,n}=|h_{1,n}|$, and $g_{0,n}=|h_{0,n}|$ represent the magnitudes of the channel coefficients, while $\omega_{U_{i}}$, i∈{1,2} denotes the additive white Gaussian noise (AWGN) with a zero mean and variance $\sigma_{U_{i}}^{2}$. The signals received in (3) can similarly be expressed as

$$y_{U_{2}}=\left(\sqrt{a_{1}P_{S}}x_{1}+\sqrt{a_{2}P_{S}}x_{2}\right)g\sum_{n=1}^{N}g_{0,n}g_{1,n}e^{j\delta_{n}}+\omega_{U_{2}},$$ (4)

Here $\delta_{n}=\vartheta +\theta_{n}+\varphi_{n}+\phi_{n}$.

### 2.2 Signal-to-interference-plus-noise ratio at two users

First, the signal-to-interference-plus-noise ratio (SINR) is calculated as the average effective signal power divided by the average noise power plus the average noise power. As a consequence, *U*_2_ detects the intended signal *x*_2_ and treats *x*_1_ as interference. The SINR for the *U*_2_ is derived as follows

$$\gamma_{U_{2},x_{2}}=\frac{\rho_{S}a_{2}{\left|g\sum_{n=1}^{N}g_{0,n}g_{1,n}e^{j\delta_{n}}\right|}^{2}}{\rho_{S}a_{1}{\left|g\sum_{n=1}^{N}g_{0,n}g_{1,n}e^{j\delta_{n}}\right|}^{2}+1},$$ (5)

where $\rho_{S}=\frac{P_{S}}{\sigma_{U_{i}}^{2}}$ is the average transmit SNR. We assume a high phase-shift resolution situation, and the technique described in [43] can be used for channel estimation. As a consequence, the ideal phase-shift design is used to optimize the SNR at the destination [44]. Let $\theta_{n}^{*}$ represent the best phase-shift of the *n*-th element of the RIS, where its value is provided by

$$\theta_{n}^{*}=-\vartheta -\phi_{n}-\varphi_{n},\forall n.$$ (6)

We rewrite the optimal instantaneous SINR at *U*_2_ as

$$\gamma_{U_{2},x_{2}}=\frac{\rho_{S}a_{2}{\left|g\sum_{n=1}^{N}g_{0,n}g_{1,n}\right|}^{2}}{\rho_{S}a_{1}{\left|g\sum_{n=1}^{N}g_{0,n}g_{1,n}\right|}^{2}+1}=\frac{a_{2}A^{2}}{a_{1}A^{2}+1},$$ (7)

where $A≜\rho_{S}\left|gG\right|$, $G≜\sum_{n=1}^{N}{𝒢}_{n}$, and ${𝒢}_{n}≜g_{0,n}g_{1,n}$.

Finally, by the NOMA downlink communication principle [45], the user *U*_1_ initially identifies *x*_2_ due to its robust signal strength, considering *x*_1_ as interference, and subsequently decodes *x*_1_ employing the SIC algorithm to eliminate *x*_2_. Consequently, the SINRs linked to *x*_2_ and *x*_1_ at *U*_1_, in scenarios of imperfect SIC are

$$\gamma_{U_{1},x_{2}}=\frac{\rho_{S}a_{2}{\left|p\right|}^{2}}{\rho_{S}a_{1}{\left|p\right|}^{2}+1},$$ (8)

$$\gamma_{U_{1},x_{1}}=\frac{\rho_{S}a_{1}{\left|p\right|}^{2}}{\zeta \rho_{S}a_{2}{\left|p\right|}^{2}+1},$$ (9)

where ζ, with 0≤ζ≤1, indicates the efficiency of SIC for *x*_2_ at the *U*_1_. The scenarios ζ=0 and ζ=1 represent pSIC and ipSIC, respectively.

## 3 Performance evaluation

### 3.1 Channel characteristics

Let us initiate our discussion by presenting the following results, which are exceptionally valuable and instrumental in deriving the intricate channel characteristics associated with the specific case of ${\left|A\right|}^{2}$.

Given a random variable, denoted as *X*, which adheres to a Nakagami-*m* distribution characterized by specific shape and scale parameters, where *m* is a natural number belonging to the set of positive integers (m∈ℕ), and Ω represents the scale parameter; additionally, there exists another random variable *Y* that conforms to a Gamma distribution defined by its parameters α and β. The cumulative distribution function (CDF) and probability density function (PDF) of the resultant random variable *Z*, which is generated by taking the product of these two random variables, specifically formulated as *Z* = *XY* is computed as

$$F_{Z}(z)=1-\sum_{v=0}^{m-1}\frac{2^{\alpha -2v-1}m^{v}{\left(\beta \right)}^{2v}z^{2v}}{\sqrt{\pi }\Gamma \left(\alpha \right)v!\Omega^{v}}\;\times G_{0,3}^{3,0}\left(\frac{{\left(\beta \right)}^{2}mz^{2}}{4\Omega }|\begin{array}{c} -\\ 0,\frac{1+\alpha -2v}{2},\frac{\alpha -2v}{2} \end{array}\right),\;f_{Z}\left(z\right)=\frac{2^{\alpha -2m}m^{m}{\left(\beta \right)}^{2m}z^{2m-1}}{\sqrt{\pi }\Omega^{m}\Gamma \left(m\right)\Gamma \left(\alpha \right)}\;\times G_{0,3}^{3,0}\left(\frac{{\left(\beta \right)}^{2}mz^{2}}{4\Omega }|\begin{array}{c} -\\ 0,\frac{1+\alpha -2m}{2},\frac{\alpha -2m}{2} \end{array}\right).$$ (10)

*Proof*: Let’s start with the following definition of the RV *Z* CDF:

$$F_{Z}(z)=\mathrm{Pr}\left(X<\frac{z}{Y}\right)\;\overset{\left(a\right)}{=}1-\sum_{v=0}^{m-1}\frac{2^{\alpha -2v-1}m^{v}{\left(\beta \right)}^{2v}z^{2v}}{\sqrt{\pi }\Gamma \left(\alpha \right)v!\Omega^{v}}\;\times G_{0,3}^{3,0}\left(\frac{{\left(\beta \right)}^{2}mz^{2}}{4\Omega }|\begin{array}{c} -\\ 0,\frac{1+\alpha -2v}{2},\frac{\alpha -2v}{2} \end{array}\right),$$ (11)

where (a) is maintained using the identity $e^{-ax}=G_{0,1}^{1,0}\left(ax|\begin{array}{c} -\\ 0 \end{array}\right)$ [46, Eq (2.6)] With the outcome from [47, Eq (2.24.1.1)], the Meijer G-function is $G_{p,q}^{m,n}\left(.\right)$ [42, Eq (9.301)]. It should be emphasized that α is a real number rather than necessarily an integer. The PDF of *Z* may therefore be easily produced by following the same procedures as the CDF:

$$f_{Z}\left(z\right)=\int_{0}^{\infty }\frac{1}{y}f_{Y}\left(y\right)f_{X}\left(\frac{z}{y}\right)dy=\frac{2^{\alpha -2m}m^{m}{\left(\beta \right)}^{2m}x^{2m-1}}{\sqrt{\pi }\Omega^{m}\Gamma \left(m\right)\Gamma \left(\alpha \right)}\;\times G_{0,3}^{3,0}\left(\frac{{\left(\beta \right)}^{2}mz^{2}}{4\Omega }|\begin{array}{c} -\\ 0,\frac{1+\alpha -2m}{2},\frac{\alpha -2m}{2} \end{array}\right).$$ (12)

The proof is completed. ◻

The following result provides the CDF and PDF of *U*_2_ after obtaining the CDF of the product of a Nakagami-*m* and a Gamma RV.

The CDF and PDF of ${\left|A\right|}^{2}$ are calculated as

$$f_{{\left|A\right|}^{2}}(x)=\frac{2^{\alpha_{G}-2m_{g}-1}m_{g}^{m_{g}}{\left(\beta_{G}\right)}^{2m_{g}}x^{m_{g}-1}}{\sqrt{\pi }\Omega_{g}^{m_{g}}\Gamma \left(m_{g}\right)\Gamma \left(\alpha_{G}\right)\rho_{S}^{m_{g}}}\;\times G_{0,3}^{3,0}\left(\frac{{\left(\beta_{G}\right)}^{2}m_{g}x}{4\rho_{S}\Omega_{g}}|\begin{array}{c} -\\ 0,\frac{1+\alpha_{G}-2m_{g}}{2},\frac{N\alpha_{G}-2m_{g}}{2} \end{array}\right),$$ (13a)

$$F_{{\left|A\right|}^{2}}(x)=1-\sum_{v=0}^{m_{g}-1}\frac{2^{\alpha_{G_{n}}-2v-1}m_{g}^{v}{\left(\beta_{G}\right)}^{2v}x^{v}}{\sqrt{\pi }\Gamma \left(\alpha_{G}\right)v!\Omega_{g}^{v}\rho_{S}^{v}}\;\times G_{0,3}^{3,0}\left(\frac{{\left(\beta_{G}\right)}^{2}m_{g}x}{4\rho_{S}\Omega_{g}}|\begin{array}{c} -\\ 0,\frac{1+\alpha_{G}-2v}{2},\frac{\alpha_{G}-2v}{2} \end{array}\right),$$ (13b)

where *m*_*g*_ and $\Omega_{g}$ are the shape and scale parameters at BD. Here Γ(.) is the Gamma function [42, Eq (8.310)], $\alpha_{𝒢}$ and $\beta_{𝒢}$ are defined in (16).

*Proof*: We start the proof by calculating the PDF of the RV ${𝒢}_{n}=g_{0,n}g_{1,n},n\in \left[1,N\right]$ as follows:

$$f_{G_{n}}\left(x\right)=\int {\;}_{0}^{\infty }\frac{1}{y}f_{g_{0,n}}\left(y\right)f_{g_{1,n}}\left(\frac{x}{y}\right)dy\;\overset{\left(a\right)}{=}\frac{4x^{m_{g_{1}}+m_{g_{0}}-1}}{\Gamma \left(m_{g_{0}}\right)\Gamma \left(m_{g_{1}}\right)}{\left(\sqrt{\frac{m_{g_{0}}m_{g_{1}}}{\Omega_{g_{0}}\Omega_{g_{1}}}}\right)}^{m_{g_{0}}+m_{g_{1}}}\;\times K_{m_{g_{0}}-m_{g_{1}}}\left(2\sqrt{\frac{m_{g_{0}}m_{g_{1}}}{\Omega_{g_{0}}\Omega_{g_{1}}}}x\right),$$ (14)

where $K_{n}\left(.\right)$ is the modified Bessel function of the second type with *n*-th order, and (a) is obtained by applying the PDF of the Nakagami-*m* RV combining with [42, Eq (3.471.9)]. Here, we assume that $m_{g_{0}}=m_{g_{0,n}}$ and $m_{g_{1}}=m_{g_{1,n}}$. $\Omega_{g_{0}}=𝔼\left\{g_{0,n}^{2}\right\}$, $\Omega_{g_{1}}=𝔼\left\{g_{1,n}^{2}\right\},\forall n$ represents the average power of channels *g*_0,*n*_ and *g*_1,*n*_. Using the PDF of $G_{n}$, we can calculate the *k*-th instant as follows:

$$\mu_{G_{n}}\left(k\right)=\int {\;}_{0}^{\infty }x^{k}f_{G_{n}}\left(x\right)dx$$ (15)

$$=\frac{\Gamma \left(m_{g_{0}}+\frac{k}{2}\right)\Gamma \left(m_{g_{1}}+\frac{k}{2}\right)}{\Gamma \left(m_{g_{0}}\right)\Gamma \left(m_{g_{1}}\right)}{\left(\sqrt{\frac{\Omega_{g_{0}}\Omega_{g_{1}}}{m_{g_{0}}m_{g_{1}}}}\;\right)}^{k}.$$

The final equation in (10) is directly derived by utilizing the result from [42, Eq (6.561.16)]. It is evident that a precise closed-form representation of the CDF and PDF for the random variable $Z=g𝒢=g\sum_{n=1}^{N}{𝒢}_{n}$ in (5) has not yet been established for an arbitrary count of RIS elements. Therefore, we employ the moment matching technique [48] to derive the closed-form expressions for the PDF and CDF of a corresponding random variable. More specifically, let us denote ${\tilde{𝒢}}_{n}$ as an equivalent random variable of ${𝒢}_{n}$, which follows a Gamma distribution characterized by shape and scale parameters, $\alpha_{G_{n}}$ and $\beta_{G_{n}}$ defined as


$$\alpha_{G_{n}}=\frac{{\left(E\left\{G_{n}\right\}\right)}^{2}}{\text{Var}\left\{G_{n}\right\}}=\frac{{\left[\mu_{G_{n}}\left(1\right)\right]}^{2}}{\mu_{G_{n}}\left(2\right)-{\left[\mu_{G_{n}}\left(1\right)\right]}^{2}},$$

$$\beta_{G_{n}}=\frac{E\left\{G_{n}\right\}}{\text{Var}\left\{G_{n}\right\}}=\frac{\mu_{G_{n}}\left(1\right)}{\mu_{G_{n}}\left(2\right)-{\left[\mu_{G_{n}}\left(1\right)\right]}^{2}}.$$ (16)

The RV $G=\sum_{n=1}^{N}G_{n}=\sum_{n=1}^{N}{\tilde{G}}_{n}$ represents the total of *N* independent and identically distributed Gamma random variables, and its PDF and CDF are consequently defined as follows


$$f_{G}\left(x,\alpha_{G},\beta_{G}\right)=\frac{{\left(\beta_{G}\right)}^{\alpha_{G}}x^{\alpha_{G}-1}e^{-\beta_{G}x}}{\Gamma \left(\alpha_{G}\right)},$$

$$F_{G}\left(x,\alpha_{G},\beta_{G}\right)=\frac{\gamma \left(\alpha_{G},\beta_{G}x\right)}{\Gamma \left(\alpha_{G}\right)},$$ (17)

where γ(·,·) is the lower incomplete Gamma function [42, Eq (8.350.2)], $\alpha_{G}=N\alpha_{G_{n}}$ and $\beta_{G}=\beta_{G_{n}},\forall n$. The CDF and PDF of ${\left|A\right|}^{2}$ as specified in (13) and (14), can be readily derived by utilizing the results from above along with the following properties: $F_{{\left|A\right|}^{2}}\left(x\right)=F_{Z}\left(\sqrt{\frac{x}{\rho_{S}}}\right)$ and $f_{{\left|A\right|}^{2}}\left(x\right)=\frac{1}{2\sqrt{\rho_{S}x}}f_{Z}\left(\sqrt{\frac{x}{\rho_{S}}}\right)$. ◻

Additionally, the PDF and CDF of ${\left|p\right|}^{2}$ follow Gamma distributions [49] with $f_{{\left|p\right|}^{2}}\left(x\right)=\frac{\mu_{p}^{m_{p}}x^{m_{p}-1}}{\Gamma \left(m_{p}\right)}e^{-\mu_{p}x}$ and $F_{{\left|p\right|}^{2}}\left(x\right)=\frac{\gamma \left(m_{p},\mu_{p}x\right)}{\Gamma \left(m_{i}\right)}$ in which $\mu_{p}=\frac{m_{p}}{\Omega_{p}}$.

### 3.2 Background of blocklength error rate

In traditional channel coding theory, the error probability of a communication system is typically examined under the assumption of infinite blocklength. Nonetheless, in real-world applications, the data rate of the system might be constrained by a predetermined finite blocklength to achieve the desired error probability. As stated in [50], the data rate *r*_*i*_, i∈{1,2} for a finite blocklength L can be estimated as

$$r_{i}\left(L,{\bar{\gamma }}_{i},{\;}_{i}\right)\approx C\left({\bar{\gamma }}_{i}\right)-\sqrt{\frac{V\left({\bar{\gamma }}_{i}\right)}{L}}Q^{-1}\left(\epsilon_{i}\right)+O\left(\frac{\log_{2}L}{L}\right),$$ (18)

where $C\left({\bar{\gamma }}_{i}\right)=\log_{2}\left(1+{\bar{\gamma }}_{i}\right)$ is the Shannon capacity, ${\bar{\gamma }}_{i}\in \left\{\gamma_{U_{2},x_{2}},\gamma_{U_{1},x_{2}},\gamma_{U_{1},x_{1}}\right\}$, $V\left({\bar{\gamma }}_{i}\right)=\left[1-{\left(1+{\bar{\gamma }}_{i}\right)}^{-2}\right]\left(\log_{2}e\right){\;}^{2}$ indicates channel dispersion (measured by squared information units per channel use) relative to a deterministic channel with the same capacity, whereas $\epsilon_{i}$ is the predicted error probability, $Q^{-1}\left(.\right)$ is the inverse Gaussian Q-function with $Q\left(x\right)=\left(2\pi \right){\;}^{-1}\int_{x}^{\infty }e^{-\frac{t^{2}}{2}}dt$ and $O\left(\frac{\log_{2}L}{L}\right)$ is the remainder term of order $\frac{\log_{2}L}{L}$. With a blocklength of L≥100, as specified in [50], we may rewrite the instantaneous BLER as

$$\epsilon_{i}\approx Q\left(\frac{C\left({\bar{\gamma }}_{i}\right)-r_{i}}{\sqrt{L^{-1}V\left({\bar{\gamma }}_{i}\right)}}\right).$$ (19)

Let $\phi_{U_{1},x_{i}}$ be the event when *x*_*i*_’s decoding mistake occurs at node *U*_1_, and ${\bar{\phi }}_{U_{1},x_{i}}$ be the complement of $\phi_{U_{1},x_{i}}$. Based on (8) and (9), the instantaneous BLER while decoding *x*_2_ at the *U*_1_ is estimated as

$$\mathrm{Pr}\left(\phi_{U_{1},x_{2}}\right)=\epsilon_{U_{1},x_{2}}\approx Q\left(\frac{C\left(\gamma_{U_{1},x_{2}}\right)-r_{2}}{\sqrt{L^{-1}V\left(\gamma_{U_{1},x_{2}}\right)}}\right),$$ (20)

where $\gamma_{U_{1},x_{2}}$ is specified in (8) and $r_{2}=\frac{B_{2}}{L}$ with *B*_2_ representing the total bit count of *x*_2_. Once the *U*_1_ successfully decodes and eliminates *x*_2_ in (2), the instantaneous BLER while decoding *x*_1_ at the *U*_1_ is

$$\mathrm{Pr}\left(\phi_{U_{1},x_{1}}|{\bar{\phi }}_{U_{1},x_{2}}\right)=\epsilon_{U_{1},x_{1}}\approx Q\left(\frac{C\left(\gamma_{U_{1},x_{1}}\right)-r_{1}}{\sqrt{L^{-1}V\left(\gamma_{U_{1},x_{1}}\right)}}\right),$$ (21)

where $\gamma_{U_{1},x_{1}}$ is specified in (9) and $r_{1}=\frac{B_{1}}{L}$ with *B*_1_ representing the total bit count of *x*_1_. Conversely, the *U*_1_ is capable of decoding *x*_1_ in the scenario where there is a SIC error at *x*_2_, denoted as $\mathrm{Pr}\left(\phi_{U_{1},x_{1}}|\phi_{U_{1},x_{2}}\right)$. Therefore, the likelihood of an error in decoding *x*_1_ at the *U*_1_ is represented as

$$\mathrm{Pr}\left(\phi_{U_{1},x_{1}}\right)=\mathrm{Pr}\left(\phi_{U_{1},x_{1}}|\phi_{U_{1},x_{2}}\right)\mathrm{Pr}\left(\phi_{U_{1},x_{2}}\right)\;+\mathrm{Pr}\left(\phi_{U_{1},x_{1}}|{\bar{\phi }}_{U_{1},x_{2}}\right)\mathrm{Pr}\left({\bar{\phi }}_{U_{1},x_{2}}\right),$$ (22)

where $\mathrm{Pr}\left(\phi_{U_{1},x_{1}}|\phi_{U_{1},x_{2}}\right)$ denotes the conditional probability of $\phi_{U_{1},x_{1}}$ given $\phi_{U_{1},x_{2}}$. Owing to the significant interference from *x*_2_ during the decoding of *x*_1_, we find that $\mathrm{Pr}\left(\phi_{U_{1},x_{1}}|\phi_{U_{1},x_{2}}\right)$ is approximately equal to 1. Based on (22), the overall BLER when identifying *x*_1_ at the *U*_1_ is determined as

$${\tilde{\epsilon }}_{U_{1},x_{1}}=1\times \epsilon_{U_{1},x_{2}}+\epsilon_{U_{1},x_{1}}\left(1-\epsilon_{U_{1},x_{2}}\right)\;=\epsilon_{U_{1},x_{2}}+\epsilon_{U_{1},x_{1}}-\epsilon_{U_{1},x_{1}}\epsilon_{U_{1},x_{2}}\;\approx \epsilon_{U_{1},x_{2}}+\epsilon_{U_{1},x_{1}}.$$ (23)

It is important to observe that in (23), the error in URLLC typically remains low, falling between 10^−3^ and 10^−5^. Hence, the term $\epsilon_{U_{1},x_{1}}\epsilon_{U_{1},x_{2}}\to 0$ can be disregarded.

On the other hand, since *U*_2_ directly interprets its message, the typical BLER of *U*_2_, denoted as $\epsilon_{U_{2},x_{2}}$, is solely dependent on its capability to recover *x*_2_ and is therefore expressed as

$${\tilde{\epsilon }}_{U_{2},x_{2}}=\epsilon_{U_{2},x_{2}},$$ (24)

where ϵU2,x2≈Q(C(γU2,x2)−r2V(γU2,x2)/V(γU2,x2)LL).

### 3.3 Average BLER in finite blocklength regime

Based on (19), the average BLER at each device may be expressed as

$${\bar{\epsilon }}_{i}\approx \int_{0}^{\infty }Q\left(\frac{C\left({\bar{\gamma }}_{i}\right)-r_{i}}{\sqrt{L^{-1}V\left({\bar{\gamma }}_{i}\right)}}\right)f_{{\bar{\gamma }}_{i}}\left(x\right)dx,$$ (25)

where $f_{{\bar{\gamma }}_{i}}\left(x\right)$ represents the PDF of the random variable ${\bar{\gamma }}_{i}$.

Because it is difficult to determine the correct closed-form expression of (25), we apply the approximation Q-function to solve (25) as comparable to [51], and [52], i.e.

$$Q\left(\frac{C\left({\bar{\gamma }}_{i}\right)-r_{i}}{\sqrt{L^{-1}V\left({\bar{\gamma }}_{i}\right)}}\right)=\{\begin{array}{cc} 1, & {\bar{\gamma }}_{i}\leq \theta_{i},\\ 0.5-\chi_{i}\left({\bar{\gamma }}_{i}-\gamma_{th,i}\right) & \theta_{i}<{\bar{\gamma }}_{i}<\varpi_{i},\\ 0, & {\bar{\gamma }}_{i}\geq \varpi_{i}, \end{array}$$ (26)

Here $\chi_{i}={\left[2\pi L^{-1}\left(2^{2r_{i}}-1\right)\right]}^{-1/2}$, $\gamma_{th,i}=2^{2r_{i}}-1$, $\theta_{i}=\gamma_{th,i}-\left(2\chi_{i}\right){\;}^{-1}$ and $\varpi_{i}=\gamma_{th,i}+\left(2\chi_{i}\right){\;}^{-1}$.

Based on (26) and (25), we may rewrite the average BLER by

$${\bar{\epsilon }}_{i}\left(x\right)\approx \chi_{i}\int_{\theta_{i}}^{\varpi_{i}}F_{{\bar{\gamma }}_{i}}\left(x\right)dx,$$ (27)

where $F_{{\bar{\gamma }}_{i}}\left(x\right)$ is the conditional CDF of ${\bar{\gamma }}_{i}$. To determine the closed-form of BLER, we first compute $F_{{\bar{\gamma }}_{i}}\left(x\right)$, which is dependent on the SINRs.

At this point, the primary objective is to establish the BLER values ${\bar{\epsilon }}_{U_{1},x_{2}}$ and ${\bar{\epsilon }}_{U_{1},x_{1}}$. Based on (23) and (27), the closed-form expression of the average BLERs of *U*_1_ is

$${\bar{\epsilon }}_{U_{1}}=𝔼\left\{{\tilde{\epsilon }}_{U_{1},x_{1}}\right\}\approx {\bar{\epsilon }}_{U_{1},x_{2}}+{\bar{\epsilon }}_{U_{1},x_{1}},$$ (28)

where ${\bar{\epsilon }}_{U_{1},x_{2}}$ and ${\bar{\epsilon }}_{U_{1},x_{1}}$ are, correspondingly, specified as

$${\bar{\epsilon }}_{U_{1},x_{2}}=\frac{\rho_{S}a_{2}\chi_{2}}{\Gamma \left(m_{p}\right)}\sum_{w_{1}=0}^{\infty }\frac{{\left(-1\right)}^{w_{1}}\mu_{p}^{m_{p}+w_{1}}}{w_{1}!\left(m_{p}+w_{1}+1\right)\left(m_{p}+w_{1}\right)}\;\times [{\bar{\varpi }}_{2}^{m_{p}+w_{1}+1}{\;}_{2}F_{1}\left(2,m_{p}+w_{1}+1;m_{p}+w_{1}+2;-\rho_{S}a_{1}{\bar{\varpi }}_{2}\right)\;-{\bar{\theta }}_{2}^{m_{p}+w_{1}+1}{\;}_{2}F_{1}\left(2,m_{p}+w_{1}+1;m_{p}+w_{1}+2;-\rho_{S}a_{1}{\bar{\theta }}_{2}\right)],$$ (29)

and

$${\bar{\epsilon }}_{U_{1},x_{1}}=\frac{\rho_{S}a_{1}\chi_{1}}{\Gamma \left(m_{p}\right)}\sum_{w_{2}=0}^{\infty }\frac{{\left(-1\right)}^{w_{2}}\mu_{p}^{m_{p}+w_{2}}}{w_{2}!\left(m_{p}+w_{2}+1\right)\left(m_{p}+w_{2}\right)}\;\times [{\bar{\varpi }}_{1}^{m_{p}+w_{2}+1}{\;}_{2}F_{1}\left(2,m_{p}+w_{2}+1;m_{p}+w_{2}+2;-\rho_{S}a_{2}\zeta {\bar{\varpi }}_{1}\right)\;-{\bar{\theta }}_{1}^{m_{p}+w_{2}+1}{\;}_{2}F_{1}\left(2,m_{p}+w_{2}+1;m_{p}+w_{2}+2;-\rho_{S}a_{2}\zeta {\bar{\theta }}_{1}\right)],$$ (30)

where ${\bar{\varpi }}_{2}=\frac{\varpi_{2}}{\rho_{S}\left(a_{2}-a_{1}\varpi_{2}\right)}$, ${\bar{\theta }}_{2}=\frac{\theta_{2}}{\rho_{S}\left(a_{2}-a_{1}\theta_{2}\right)}$, ${\bar{\varpi }}_{1}=\frac{\varpi_{1}}{\rho_{S}\left(a_{2}-a_{2}\zeta \varpi_{1}\right)}$ and ${\bar{\theta }}_{1}=\frac{\theta_{1}}{\rho_{S}\left(a_{2}-a_{2}\zeta \theta_{1}\right)}$.

*Proof*: To begin with, the CDFs of $\gamma_{U_{1},x_{2}}$ and $\gamma_{U_{1},x_{1}}$ are expressed as follows:

$$F_{\gamma_{U_{1},x_{2}}}\left(x\right)=F_{{\left|p\right|}^{2}}\left(\nu \left(x\right)\right)=\frac{\gamma \left(m_{p},\mu_{p}\nu \left(x\right)\right)}{\Gamma \left(m_{p}\right)},\;\text{and},$$ (31)

$$F_{\gamma_{U_{1},x_{1}}}\left(x\right)=F_{{\left|p\right|}^{2}}\left(\eta \left(x\right)\right)=\frac{\gamma \left(m_{p},\mu_{p}\eta \left(x\right)\right)}{\Gamma \left(m_{p}\right)},$$ (32)

where $\nu \left(x\right)=\frac{x}{\rho_{S}\left(a_{2}-a_{1}x\right)}$ and $\eta \left(x\right)=\frac{x}{\rho_{S}\left(a_{1}-a_{2}\zeta x\right)}$. Also, (31) and (32) are obtained under the condition $a_{2}-a_{1}x>0$ and $a_{1}-a_{2}\zeta x>0$, respectively.

By substituting (31) into (27) and (32) into (27), the average BLER for decoding *x*_2_ and *x*_1_ at *U*_1_ is obtained as follows

$${\bar{\epsilon }}_{U_{1},x_{2}}=\chi_{2}\int_{\theta_{2}}^{\varpi_{2}}\frac{\gamma \left(m_{p},\mu_{p}\nu \left(x\right)\right)}{\Gamma \left(m_{p}\right)}dx,$$ (33)

$${\bar{\epsilon }}_{U_{1},x_{1}}=\chi_{1}\int_{\theta_{1}}^{\varpi_{1}}\frac{\gamma \left(m_{p},\mu_{p}\eta \left(x\right)\right)}{\Gamma \left(m_{p}\right)}dx.$$ (34)

Now, by taking a variable change $z_{1}=\frac{x}{\rho_{S}\left(a_{2}-a_{1}x\right)}$ and $z_{2}=\frac{x}{\rho_{S}\left(a_{1}-a_{2}\zeta x\right)}$, ${\bar{\epsilon }}_{U_{1},x_{2}}$ and ${\bar{\epsilon }}_{U_{1},x_{1}}$ are rewritten as

$${\bar{\epsilon }}_{U_{1},x_{2}}=\frac{\rho_{S}a_{2}\chi_{2}}{\Gamma \left(m_{p}\right)}\int_{{\bar{\theta }}_{2}}^{{\bar{\varpi }}_{2}}\frac{\gamma \left(m_{p},\mu_{p}z_{1}\right)}{{\left(1+\rho_{S}a_{1}z_{1}\right)}^{2}}dz_{1},$$ (35)

$${\bar{\epsilon }}_{U_{1},x_{1}}=\frac{\rho_{S}a_{1}\chi_{1}}{\Gamma \left(m_{p}\right)}\int_{{\bar{\theta }}_{1}}^{{\bar{\varpi }}_{1}}\frac{\gamma \left(m_{p},\mu_{p}z_{2}\right)}{{\left(1+\rho_{S}a_{2}\zeta z_{2}\right)}^{2}}dz_{2},$$ (36)

where ${\bar{\varpi }}_{2}=\frac{\varpi_{2}}{\rho_{S}\left(a_{2}-a_{1}\varpi_{2}\right)}$, ${\bar{\theta }}_{2}=\frac{\theta_{2}}{\rho_{S}\left(a_{2}-a_{1}\theta_{2}\right)}$, ${\bar{\varpi }}_{1}=\frac{\varpi_{1}}{\rho_{S}\left(a_{2}-a_{2}\zeta \varpi_{1}\right)}$ and ${\bar{\theta }}_{1}=\frac{\theta_{1}}{\rho_{S}\left(a_{2}-a_{2}\zeta \theta_{1}\right)}$.

With the help [42, Eq (8.354.1)], ${\bar{\epsilon }}_{U_{1},x_{2}}$ and ${\bar{\epsilon }}_{U_{1},x_{1}}$ are derived as

$${\bar{\epsilon }}_{U_{1},x_{2}}=\frac{\rho_{S}a_{2}\chi_{2}}{\Gamma \left(m_{p}\right)}\sum_{w_{1}=0}^{\infty }\frac{{\left(-1\right)}^{w_{1}}\mu_{p}^{m_{p}+w_{1}}}{w_{1}!\left(m_{p}+w_{1}\right)}\int_{{\bar{\theta }}_{2}}^{{\bar{\varpi }}_{2}}\frac{z_{1}^{m_{p}+w_{1}}}{{\left(1+\rho_{S}a_{1}z_{1}\right)}^{2}}dz_{1},$$ (37)

$${\bar{\epsilon }}_{U_{1},x_{1}}=\frac{\rho_{S}a_{1}\chi_{1}}{\Gamma \left(m_{p}\right)}\sum_{w_{2}=0}^{\infty }\frac{{\left(-1\right)}^{w_{2}}\mu_{p}^{m_{p}+w_{2}}}{w_{2}!\left(m_{p}+w_{2}\right)}\int_{{\bar{\theta }}_{1}}^{{\bar{\varpi }}_{1}}\frac{z_{2}^{m_{p}+w_{2}}}{{\left(1+\rho_{S}a_{2}\zeta z_{2}\right)}^{2}}dz_{2}.$$ (38)

By applying [42, Eq (3.194.1)] and following a series of derivation steps, one can arrive at a precise closed-form expression for the average BLER when retrieving *x*_1_ and *x*_2_ at *U*_*i*_ as

$${\bar{\epsilon }}_{U_{1},x_{2}}=\frac{\rho_{S}a_{2}\chi_{2}}{\Gamma \left(m_{p}\right)}\sum_{w_{1}=0}^{\infty }\frac{{\left(-1\right)}^{w_{1}}\mu_{p}^{m_{p}+w_{1}}}{w_{1}!\left(m_{p}+w_{1}+1\right)\left(m_{p}+w_{1}\right)}\;\times [{\bar{\varpi }}_{2}^{m_{p}+w_{1}+1}{\;}_{2}F_{1}\left(2,m_{p}+w_{1}+1;m_{p}+w_{1}+2;-\rho_{S}a_{1}{\bar{\varpi }}_{2}\right)\;-{\bar{\theta }}_{2}^{m_{p}+w_{1}+1}{\;}_{2}F_{1}\left(2,m_{p}+w_{1}+1;m_{p}+w_{1}+2;-\rho_{S}a_{1}{\bar{\theta }}_{2}\right)],$$ (39)

and

$${\bar{\epsilon }}_{U_{1},x_{1}}=\frac{\rho_{S}a_{1}\chi_{1}}{\Gamma \left(m_{p}\right)}\sum_{w_{2}=0}^{\infty }\frac{{\left(-1\right)}^{w_{2}}\mu_{p}^{m_{p}+w_{2}}}{w_{2}!\left(m_{p}+w_{2}+1\right)\left(m_{p}+w_{2}\right)}\;\times [{\bar{\varpi }}_{1}^{m_{p}+w_{2}+1}{\;}_{2}F_{1}\left(2,m_{p}+w_{2}+1;m_{p}+w_{2}+2;-\rho_{S}a_{2}\zeta {\bar{\varpi }}_{1}\right)\;-{\bar{\theta }}_{1}^{m_{p}+w_{2}+1}{\;}_{2}F_{1}\left(2,m_{p}+w_{2}+1;m_{p}+w_{2}+2;-\rho_{S}a_{2}\zeta {\bar{\theta }}_{1}\right)].$$ (40)

Substituting (40) and (39) into (28), we can obtain $𝔼\left\{{\tilde{\epsilon }}_{U_{1},x_{1}}\right\}$. The proof is completed. ◻

From (24) the average BLER at *U*_2_ is

$${\bar{\epsilon }}_{U_{2}}=𝔼\left\{{\tilde{\epsilon }}_{U_{2},x_{2}}\right\}={\bar{\epsilon }}_{U_{2},x_{2}}.$$ (41)

Submitting (13a) into (27), ${\bar{e}}_{U_{2},x_{2}}$ can be calculated as

$${\bar{e}}_{U_{2},x_{2}}=\chi_{2}\int_{\theta_{2}}^{\varpi_{2}}F_{{\bar{\gamma }}_{U_{2},x_{2}}}\left(x\right)dx=\chi_{2}\int_{\theta_{2}}^{\varpi_{2}}F_{{\left|A\right|}^{2}}\left(\frac{x}{a_{2}-a_{1}x}\right)\;=1-\chi_{2}\sum_{v=0}^{m_{g}-1}\frac{2^{\alpha_{G}-2v-1}m_{g}^{v}{\left(\beta_{G}\right)}^{2v}}{\sqrt{\pi }\Gamma \left(\alpha_{G}\right)v!\Omega_{g}^{v}\rho_{S}^{v}}\int_{\theta_{2}}^{\varpi_{2}}{\left(\frac{x}{a_{2}-a_{1}x}\right)}^{v}\;\times G_{0,3}^{3,0}\left(\frac{{\left(\beta_{G}\right)}^{2}m_{g}}{4\rho_{S}\Omega_{g}}\left(\frac{x}{a_{2}-a_{1}x}\right)|\begin{array}{c} -\\ 0,\frac{1+\alpha_{G}-2v}{2},\frac{\alpha_{G}-2v}{2} \end{array}\right)dx.$$ (42)

Unfortunately, finding a closed-form expression for (42) is a tough task, but an accurate approximation can be obtained for it. By using Gaussian-Chebyshev quadrature [53, Eq (25.4.38)], ${\bar{\epsilon }}_{U_{2},x_{2}}$ can be achieved

$${\bar{e}}_{U_{2},x_{2}}\approx 1-\frac{\pi \chi_{2}\left(\varpi_{2}-\theta_{2}\right)}{W\Gamma \left(\alpha_{G}\right)\sqrt{\pi }}\sum_{v=0}^{m_{g}-1}\frac{2^{\alpha_{G}-2v-2}m_{g}^{v}{\left(\beta_{G}\right)}^{2v}}{v!\Omega_{g}^{v}\rho_{S}^{v}}\;\times \sum_{w=1}^{W}\left|\sin\frac{\left(2w-1\right)\pi }{2W}\right|\frac{\psi_{w}^{v}}{{\left(a_{2}-a_{1}\psi_{w}\right)}^{v}}\;\times G_{0,3}^{3,0}\left(\frac{{\left(\beta_{G}\right)}^{2}m_{g}\psi_{w}}{4\rho_{S}\Omega_{g}\left(a_{2}-a_{1}\psi_{w}\right)}|\begin{array}{c} -\\ 0,\frac{1+\alpha_{G}-2v}{2},\frac{\alpha_{G}-2v}{2} \end{array}\right),$$ (43)

where *W* denotes the number of the Gauss-Chebyshev nodes, and $\psi_{w}=0.5\chi_{2}^{-1}\cos\left(\frac{\left(2w-1\right)\pi }{2W}\right)+\;\gamma_{th,2}$.

### 3.4 Average asymptotic BLER analysis

From (27), employing the first-order Riemann integral estimation, A can be estimated as

$${\bar{\epsilon }}_{i}\approx F_{{\bar{\gamma }}_{i}}\left(\gamma_{th,i}\right).$$ (44)

Based on (31) and (32), and applying γ(a,x)→xa/xaaa for *x* approaching 0, the mean asymptotic BLER at *U*_1_ is

$${\bar{\epsilon }}_{U_{1}}^{\infty }=\frac{\mu_{p}^{m_{p}}}{\Gamma \left(m_{p}+1\right)}\left\langle {\left[\nu \left(\gamma_{th,2}\right)\right]}^{m_{p}}+{\left[\eta \left(\gamma_{th,1}\right)\right]}^{m_{p}}\right\rangle .$$ (45)

Furthermore, the average asymptotic BLER at *U*_2_ is described by

$${\bar{\epsilon }}_{U_{2}}^{i}nfty\approx \frac{\Gamma \left(\alpha_{G}-2m_{g}\right)}{\Gamma \left(\alpha_{G}\right)\Gamma \left(m_{g}+1\right)}{\left(\frac{m_{g}}{\Omega_{g}}\right)}^{m_{g}}{\left(\frac{\beta_{G}^{2}\gamma_{{th}_{2}}}{\rho_{S}}\right)}^{m_{g}}.$$ (46)

*Proof*: We begin with the following asymptotic of the Nakagami-*m* distribution’s PDF when $\rho_{S}\to \infty$ as

$$F_{g}^{\infty }(x)\approx \frac{x^{2m_{g}}}{\Gamma \left(m_{g}+1\right)}{\left(\frac{m_{g}}{\Omega_{g}}\right)}^{m_{g}}.$$ (47)

The asymptotic behavior of the CDF of *Z* in (10) can be expressed as

$$F_{Z}^{\infty }(x)\approx \int_{0}^{\infty }f_{G}(z)F_{g}^{\infty }\left(\frac{x}{z}\right)dz={\left(\frac{m_{g}}{\Omega_{g}}\right)}^{m_{g}}\;\times \frac{x^{2m_{g}}{\left(\beta_{G}\right)}^{\alpha_{G}}}{\Gamma \left(\alpha_{G}\right)\Gamma \left(m_{g}+1\right)}\int_{0}^{\infty }z^{\alpha_{G}-2m_{g}-1}e^{-\beta_{G}z}dz\;=\frac{\Gamma \left(\alpha_{G}-2m_{g}\right){\left(\beta_{G}x\right)}^{2m_{g}}}{\Gamma \left(\alpha_{G}\right)\Gamma \left(m_{g}+1\right)}{\left(\frac{m_{g}}{\Omega_{g}}\right)}^{m_{g}}.$$ (48)

Here the last equation is attained via the help of [42, Eq (3.351.3)]. Furthermore, the asymptotic of $F_{{\left|A\right|}^{2}}\left(x\right)$ is given by (49) and we conclude the proof here.

$$\;\;F_{{\left|A\right|}^{2}}^{\infty }(x)\;\approx \;\;\frac{\Gamma \left(\alpha_{G}-2m_{g}\right)}{\Gamma \left(\alpha_{G}\right)\Gamma \left(m_{g}+1\right)}{\left(\frac{m_{g}}{\Omega_{g}}\right)}^{m_{g}}\;\;{\left(\frac{\beta_{G}^{2}x}{\rho_{S}}\right)}^{m_{g}}.$$ (49)

◻

**Remark:** Interestingly, from (46), we observe that the diversity order of the considered system heavily depends on the strength of the link between the BS and BD, characterized by the shape parameter *m*_*g*_ [54]. Specifically, a larger *m*_*g*_ leads to a lower average BLER, indicating that the system becomes more reliable when the channel conditions improve. Moreover, (46) reveals that the average BLER performance is directly influenced by the channel gain $\Omega_{g}$. Additionally, it is evident that increasing the number of RIS elements-represented by the term $\alpha_{𝒢}$-enhances the average BLER performance. This suggests that system reliability can be significantly improved by deploying more RIS elements.

### 3.5 Reliability and throughput analysis

In this section, we evaluate the reliability and throughput of the RIS-assisted AmBC system in conjunction with the NOMA system for SPC. The probability of receiving and properly decoding a broadcast signal at the destination is utilized to determine reliability. Various factors, such as the likelihood of successfully transmitting a symbol with unlimited retransmission time and the maximum number of symbols per packet, can influence a communication system’s reliability. Let $R_{U_{i}}$, i∈{1,2} represent the reliability of transmitting *x*_*i*_, expressed as a percentage (%), that is

$$R_{U_{i}}=100\times \left(1-{\bar{\epsilon }}_{U_{i}}\right).$$ (50)

When the blocklength is significantly large, the ergodic capacity is consistently employed as a benchmark for evaluation. Conversely, in cases where the blocklength is brief, the throughput is utilized instead of the ergodic capacity. In this segment, we derive the throughput for the examined RIS-Assisted Ambient Backscatter NOMA SPC system. The throughput of a short-packet communication system is characterized as the number of packets successfully decoded each second [50]. Throughput assesses the efficiency and effectiveness of data processing within the specified system. As the block error rate rises, throughput generally declines, or the opposite may occur. Therefore, the average throughput of *U*_*i*_ is expressed as [55]

$$\tau_{U_{i}}=\left(1-{\bar{\epsilon }}_{U_{i}}\right)r_{i}.$$ (51)

For the studied RIS-supported ambient backscatter NOMA SPC system, the overall throughput is calculated as

$$\tau_{sys}=\tau_{U_{1}}+\tau_{U_{2}}.$$ (52)

## 4 Numerical results

In this section, we perform a numerical analysis of our theoretical findings concerning the average BLER performance. We configure the fading parameters to $m=m_{g}=m_{g_{0}}=m_{g_{1}}=m_{p}$. The results from the Monte Carlo simulations [56–58] are averaged across 10^6^ independent trials. In the subsequent figures, we refer to “Ana.”, “Sim.”, and “Asym.” to represent analytical calculations, Monte Carlo simulations, and simulations based on asymptotic computations, respectively. The other key parameters are outlined in Table 3. Furthermore, we have chosen the Gauss-Chebyshev parameter as *W* = 100 to achieve a close approximation.

**Table 3:** Definition of system parameters.

Parameters	Notation	Values
Monte Carlo simulations	–	10^6^ iterations
The power allocation coefficient	$\left\{a_{1},a_{2}\right\}$	{0.2,0.8}
Total reflecting elements	*N*	6
The fading parameter	*m*	2
Blocklength	L	200
Number of information bits	$B_{1}=B_{2}$	80 bits
Imperfect SIC impacting factor	ζ	0.01
The average power	$\Omega_{p}$	1
	$\Omega_{g}$	0.1
	$\Omega_{g_{0}}$	0.3
	$\Omega_{g_{1}}$	0.4

**Fig 2:**
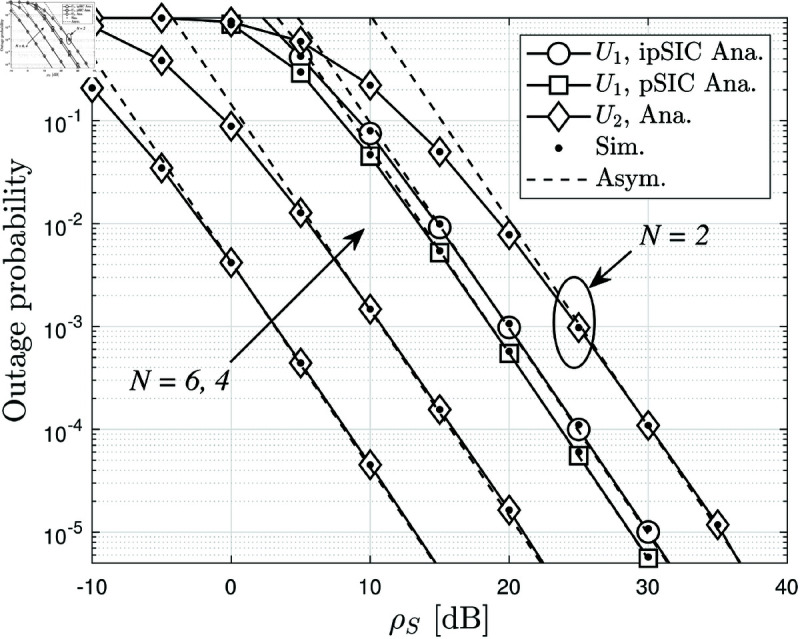
The average BLER of two users versus $\rho_{S}$ with different RIS reflector numbers with ζ=0.2.

Fig 2 illustrates the average BLER performance of *U*_1_ and *U*_2_ versus $\rho_{S}$ in dB, for different numbers of RIS reflecting elements *N*. The analytical results for *U*_1_ and *U*_2_ correspond to (28) and (41), respectively, while the simulated results for both users follow (27). Additionally, the asymptotic expressions for *U*_1_ and *U*_2_ are given by (45) and (46), respectively. The results confirm that as $\rho_{S}$ increases, the BLER of both users decreases due to improved signal quality. Furthermore, increasing *N* significantly enhances performance, particularly for *U*_2_, which experiences a weaker direct link and relies more on RIS-assisted reflection. The performance gap between *U*_1_ and *U*_2_ highlights the effectiveness of NOMA in resource allocation, where *U*_1_ benefits from direct transmission while *U*_2_ leverages both ambient backscatter and RIS. An interesting observation from Fig 2 is that when *N* = 2, *U*_2_ has a higher BLER than *U*_1_, meaning its performance is worse. This is because with a small number of reflecting elements, the RIS is not able to sufficiently enhance the signal strength for *U*_2_, and the impact of path loss dominates. However, as *N* increases beyond 2, *U*_2_ starts to outperform *U*_1_ in terms of BLER. This is due to the cumulative effect of multiple RIS elements, which significantly boost the received signal power at *U*_2_, making it more resilient to noise and fading. This trend emphasizes the critical role of RIS in balancing the performance of users in NOMA-based systems and demonstrates that sufficient RIS deployment can effectively compensate for the weaker direct link of *U*_2_. The close agreement between the analytical, simulated, and asymptotic results further validates the accuracy of our derived expressions.

**Fig 3:**
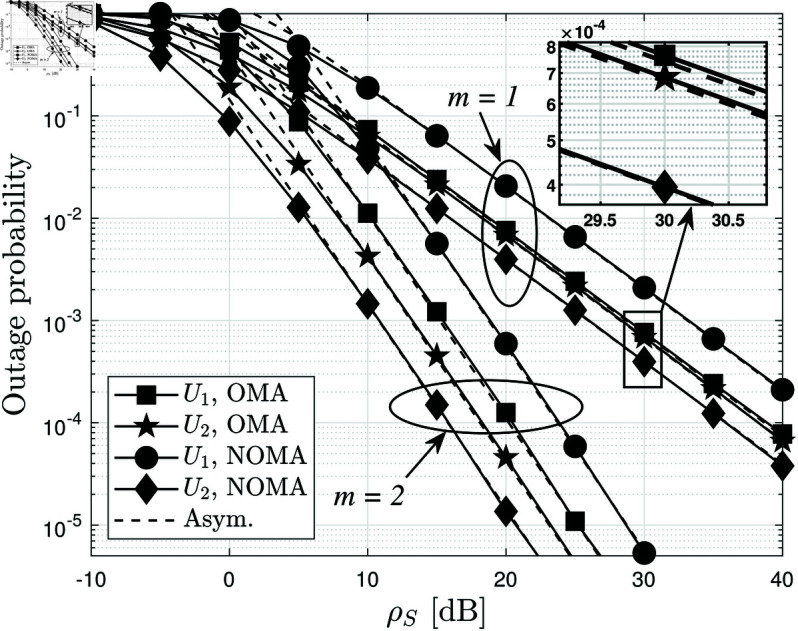
The average BLER of two users versus $\rho_{S}$ comparison between NOMA and OMA, with different m={1,2} and N=4.

Fig 3 compares the BLER performance of NOMA and OMA under different values of the fading parameter *m* versus $\rho_{S}$ in dB. The results demonstrate that NOMA consistently outperforms OMA in terms of BLER reduction, particularly in the high-SNR regime. This improvement stems from the power-domain multiplexing in NOMA, which efficiently allocates resources between users, leading to better spectrum utilization and stronger interference management. When *m* = 2, the system experiences less severe fading, resulting in lower BLER than when *m* = 1. This is because a higher *m* corresponds to improved channel conditions, reducing the probability of deep fades and enhancing signal reliability. The difference in performance between NOMA and OMA is more pronounced when *m* = 1, where fading is more severe. This suggests that NOMA is particularly beneficial in challenging wireless environments, as it provides superior robustness against fading compared to OMA. Additionally, as $\rho_{S}$ increases, the gap between NOMA and OMA widens, confirming that NOMA achieves better reliability in high-SNR conditions. This highlights the advantage of NOMA in scenarios where users experience significantly different channel conditions, as it allows efficient resource allocation while maintaining lower BLER. The results in Fig 3 reinforce the suitability of NOMA for RIS-assisted AmBC systems, especially in environments with strong fading effects. These findings further motivate integrating NOMA with RIS to maximize spectral efficiency and system robustness in next-generation wireless networks.

**Fig 4:**
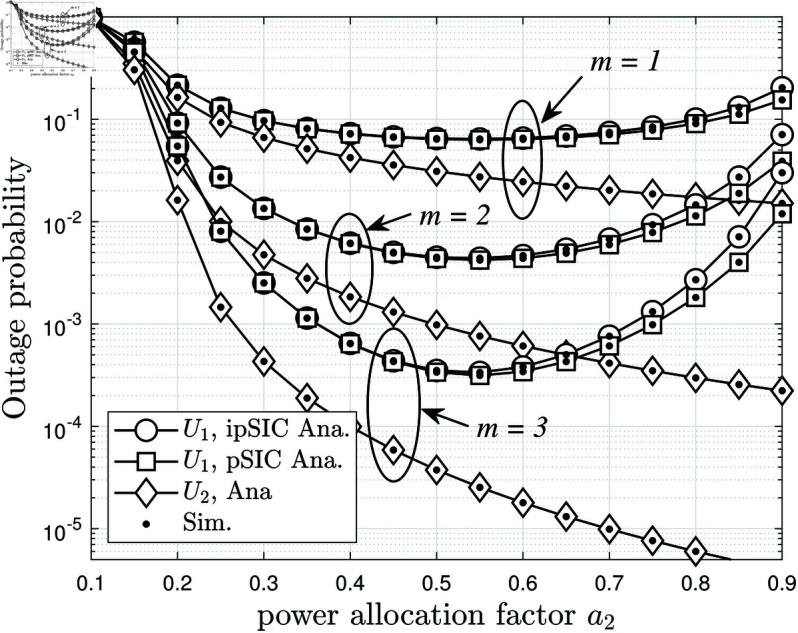
The average BLER of two users versus $a_{2}$, with different m={1,2,3}, N=4, $\rho_{S}=10$ dB and ζ=0.2.

Fig 4 presents the average BLER performance of both users as a function of the power allocation coefficient *a*_2_, under different values of *m*, while keeping *N* = 4 and $\rho_{S}=10$ dB. The results reveal key insights into the impact of power allocation on system reliability. When *a*_2_ increases, more power is allocated to the far user *U*_2_, while the near user *U*_1_ receives a lower power fraction. This causes a noticeable trade-off: the BLER of *U*_2_ gradually decreases due to improved signal strength, while the BLER of *U*_1_ worsens due to reduced power allocation. This trade-off is a fundamental characteristic of power-domain NOMA, highlighting the importance of optimizing *a*_2_ to balance performance between both users. The impact of *m*, which represents the fading severity, is also evident. When *m* = 1, corresponding to more severe fading conditions, the overall BLER is significantly higher for both users. In contrast, when *m* = 3, the fading effects are mitigated, leading to substantial BLER reduction. This demonstrates that improving channel conditions (i.e., higher *m*) enhances the overall system reliability. Interestingly, for small values of *a*_2_, *U*_1_ outperforms *U*_2_ in terms of BLER due to its stronger direct link and higher initial power allocation. However, as *a*_2_ increases, *U*_2_ gradually achieves better BLER performance, eventually surpassing *U*_1_ when *a*_2_ is large enough. This emphasizes the importance of carefully selecting *a*_2_ based on system requirements, ensuring a balance between user fairness and overall BLER minimization. These results further confirm the flexibility of NOMA in resource allocation and its ability to adapt to different fading conditions.

**Fig 5:**
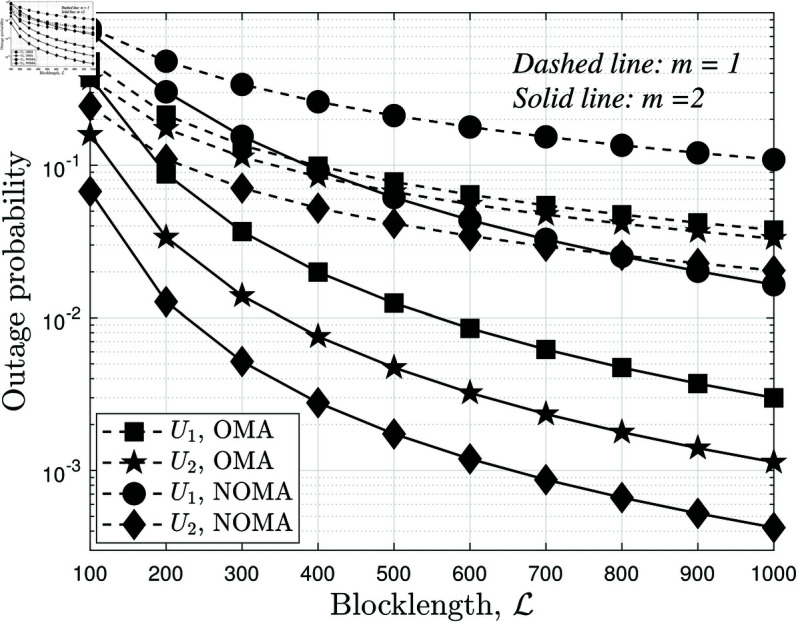
The average BLER of two users versus blocklength L, with different m={1,2}, N=4 and $\rho_{S}=5$ dB.

Fig 5 illustrates the impact of blocklength L on the average BLER performance of both users under different values of *m* while keeping *N* = 4 and $\rho_{S}=5$ dB. As expected, the BLER decreases as L increases. This is because a larger blocklength allows more redundancy in the transmitted packets, improving error correction capability and enhancing decoding reliability. However, this improvement follows a diminishing return effect, where increasing L beyond a certain threshold results in only marginal gains. This highlights the trade-off between latency and reliability in SPC: while longer blocklengths reduce BLER, they also increase transmission delay, which may not be suitable for URLLC scenarios. The effect of the fading parameter *m* is also evident. When *m* = 1, indicating a more severe fading environment, the overall BLER is significantly higher compared to *m* = 2. This confirms that better channel conditions (higher *m*) improve the reliability of short-packet transmission. Additionally, the gap between the curves for different *m* values widens as L increases, suggesting that fading severity has a stronger impact when blocklength is small. This implies that for SPC applications operating under severe fading, relying on additional diversity techniques (e.g., increasing RIS elements or optimizing power allocation) becomes even more critical. Furthermore, the comparison between *U*_1_ and *U*_2_ shows that *U*_1_ consistently achieves lower BLER than *U*_2_ across all blocklength values. This is expected since *U*_1_ benefits from a direct link with the base station, whereas *U*_2_ relies on RIS and backscatter communication, making it more susceptible to fading and interference. However, as L increases, the performance gap between the two users narrows, indicating that longer blocklengths help mitigate the disadvantage of the weaker link at *U*_2_. A key observation in Fig 5 is the comparison between NOMA and OMA. Across all blocklength values, NOMA consistently achieves lower BLER than OMA, demonstrating its superiority in short-packet transmission. This performance gain is attributed to the efficient power-domain multiplexing in NOMA, which allows better resource allocation between users, whereas OMA suffers from spectral inefficiency due to fixed bandwidth partitioning. The advantage of NOMA becomes even more pronounced when L is small, where its interference management capability significantly reduces decoding errors compared to OMA. However, as L increases, the BLER gap between NOMA and OMA narrows, indicating that the benefits of NOMA are most prominent in ultra-short-packet scenarios.

**Fig 6:**
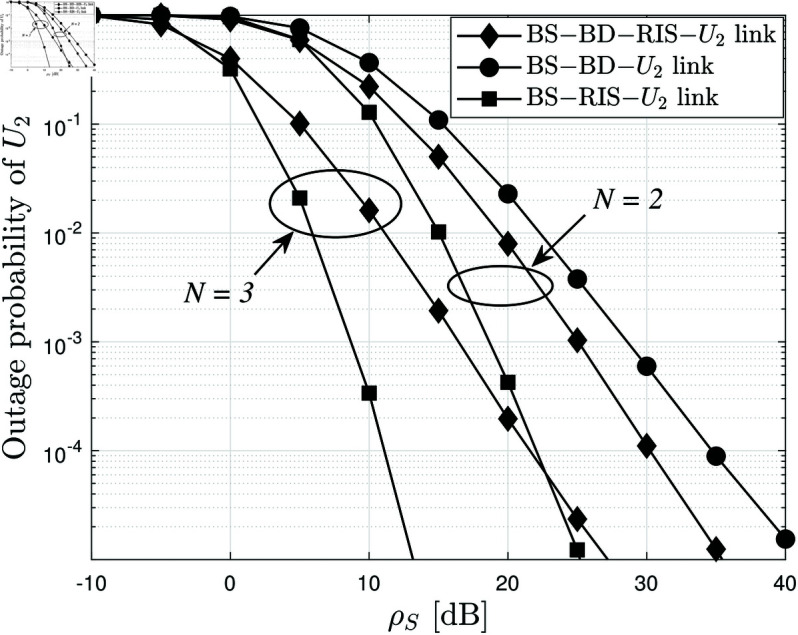
The average BLER of $U_{2}$ versus $\rho_{S}$, with different m=2 and N=2,3.

Fig 6 clearly shows three distinct curves for *U*_2_’s outage probability under the proposed RIS-AmBC-NOMA system (BS–BD–RIS−*U*_2_), the AmBC-only link (BS–BD−*U*_2_), and the RIS-only link (BS–RIS−*U*_2_), each plotted for *N* = 2 and *N* = 3. The combined RIS-AmBC path yields the lowest outage across the SNR range, confirming the synergy between ambient backscatter diversity and RIS reflection. The RIS-only curve sits in the middle, demonstrating that RIS alone substantially reduces outage compared to AmBC-only, but cannot match the full system’s performance. Conversely, the AmBC-only curve exhibits the highest outage, especially at low to moderate SNRs, highlighting that backscatter alone provides limited gain without RIS assistance. As *N* increases from 2 to 3, all three curves shift leftward, reinforcing that more reflecting elements uniformly enhance reliability. The spread between the three curves at a given *N* quantifies each component’s individual contribution: RIS yields a larger improvement over the BD-only link, while the combination of both yields the greatest gain. Overall, the figure robustly confirms that neither RIS nor AmBC alone suffices for ultra-reliable short-packet transmission, but their integration drives the most significant BLER reduction.

**Fig 7:**
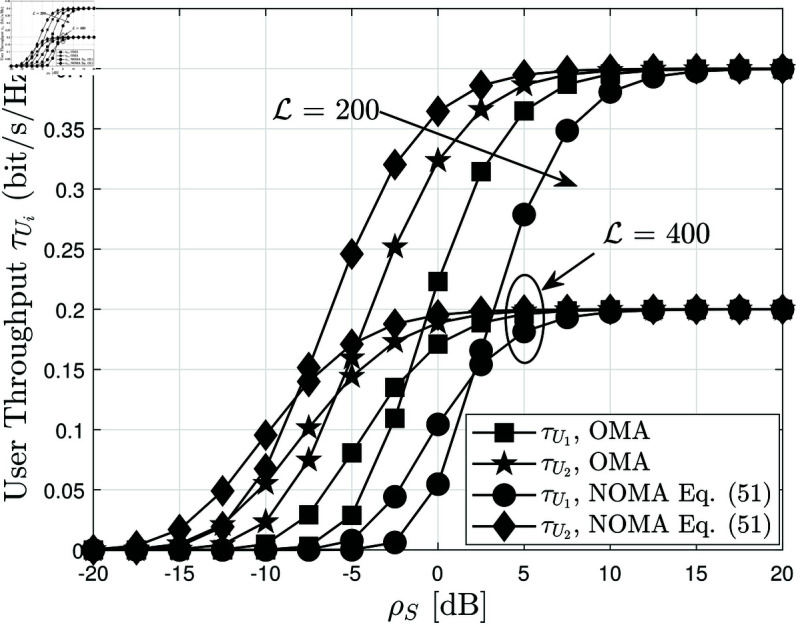
The reliability versus $\rho_{S}$, with different L = 300, N=4 and m=2.

Fig 7 illustrates the system reliability as a function of $\rho_{S}$, the average SNR, under different blocklength values L=300, while keeping *N* = 4 and *m* = 2. Reliability is a crucial metric in SPC, reflecting the probability that a transmitted packet is successfully decoded. As expected, reliability improves as $\rho_{S}$ increases, since a higher SNR enhances signal reception and reduces decoding errors. This trend is consistent with previous BLER results, where increasing $\rho_{S}$ leads to lower error rates, thereby improving the probability of successful packet decoding. Additionally, the figure highlights the impact of L on reliability. Since larger blocklengths provide more redundancy and better error correction, reliability improves significantly when L increases. However, this comes at the cost of increased transmission delay, emphasizing the trade-off between reliability and latency in SPC. Another key observation is the performance difference between NOMA and OMA. Across all SNR values, NOMA consistently achieves higher reliability than OMA, reinforcing its advantage in short-packet communication. This performance gain is attributed to NOMA’s power-domain multiplexing, which efficiently manages interference and allocates power to maximize successful packet decoding. In contrast, OMA suffers from spectral inefficiency, leading to lower reliability, especially in the low-SNR regime. However, as $\rho_{S}$ increases, the reliability gap between NOMA and OMA narrows, suggesting that OMA performs relatively better under high-SNR conditions where interference is less dominant. These findings further validate the effectiveness of NOMA in enhancing system reliability for SPC applications.

**Fig 8:**
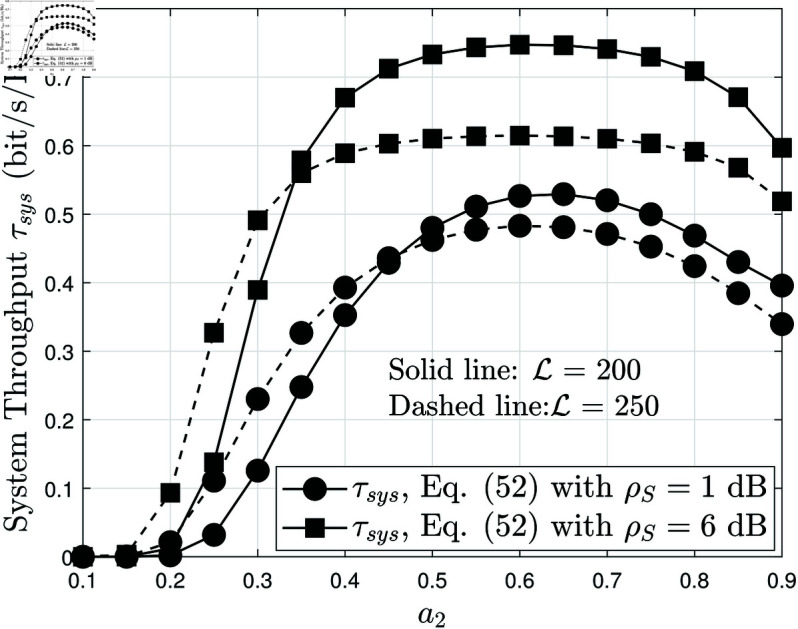
Throughput of $U_{i}$ versus $\rho_{S}$, with different L={200,400}, N=4 and m=2.

Fig 8 depicts the system throughput of user *U*_*i*_ versus $\rho_{S}$, the average SNR, for different blocklength values L={200,400}, while keeping *N* = 4 and *m* = 2. Throughput is a critical performance metric in SPC, reflecting the number of successfully decoded packets per second. As expected, throughput increases as $\rho_{S}$ grows, since a higher SNR leads to lower BLER, allowing more packets to be correctly received and decoded. However, the rate of increase diminishes at high $\rho_{S}$, indicating that throughput gains become less significant once the BLER is sufficiently low. The impact of blocklength L is also evident. A larger L improves error correction, reducing BLER and consequently enhancing throughput. However, this comes at the cost of increased latency, as longer packets require more transmission time. The results highlight a fundamental trade-off in SPC: while increasing L improves decoding reliability, it may also limit the number of packets transmitted per unit time, affecting overall system efficiency. A key observation in Fig 8 is the comparison between NOMA and OMA. Across all SNR values, NOMA consistently achieves higher throughput than OMA, reinforcing its advantage in SPC. The performance gap between NOMA and OMA is more pronounced at low $\rho_{S}$, where NOMA’s interference management capability provides a significant advantage. However, $\rho_{S}$ increases, the gap narrows since OMA benefits from improved channel conditions and reduced interference. These results emphasize the importance of optimizing L and power allocation strategies to maximize throughput in RIS-assisted AmBC-NOMA systems.

**Fig 9:**
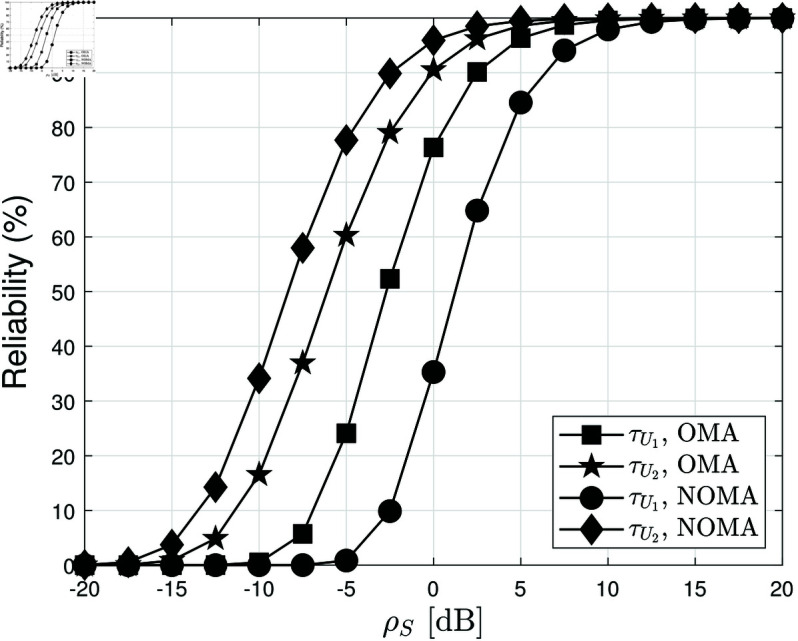
System throughput versus $\rho_{S}$, with different L={200,250}, N=4 and m=2.

Fig 9 illustrates the system throughput as a function of $\rho_{S}$, the average SNR, for different blocklength values L={200,400}, while keeping *N* = 4 and *m* = 2. This figure provides insights into how blocklength and SNR affect overall system efficiency in RIS-assisted AmBC-NOMA networks. As observed in previous results, throughput increases as $\rho_{S}$ grows, since a higher SNR leads to lower BLER, allowing more successfully decoded packets per unit time. However, similar to Fig 8, the rate of increase diminishes at high $\rho_{S}$, suggesting that once the BLER is sufficiently low, further improvements in SNR yield only marginal throughput gains. The impact of blocklength L is also evident. When L increases from 200 to 250, the system achieves better error correction, reducing BLER and improving the probability of successful packet transmission. Consequently, the throughput for L=250 is higher than for L=200. However, the gap between the two curves narrows at high $\rho_{S}$, indicating that the benefits of increasing L become less significant when the channel conditions are strong. This highlights a key trade-off in short-packet communications: increasing L enhances decoding accuracy but also introduces additional transmission delay, which may not be desirable in URLLC scenarios. Another critical observation is the performance comparison between NOMA and OMA. Across all SNR values, NOMA consistently achieves higher throughput than OMA, reinforcing its superiority in resource allocation and spectral efficiency. This is because NOMA allows simultaneous transmission to multiple users in the power domain, whereas OMA divides resources between users, leading to lower overall throughput. The gap between NOMA and OMA is most pronounced at low $\rho_{S}$, where NOMA’s interference management provides a significant advantage. However, as $\rho_{S}$ increases, the difference between the two schemes reduces, as OMA benefits from improved channel conditions and reduced interference. These findings highlight the importance of optimizing blocklength and resource allocation strategies to maximize throughput in RIS-assisted AmBC-NOMA systems.

## 5 Conclusion

This paper analyzed the BLER performance of a RIS-assisted AmBC system with NOMA under Nakagami-m fading. We derived closed-form expressions for the average and asymptotic BLER, providing insights into how system parameters, such as the number of RIS elements and power allocation, affect reliability. Our results demonstrated that RIS significantly enhances signal reception, reducing BLER and improving throughput, making it a promising technology for SPC in URLLC scenarios. Numerical and Monte Carlo simulations validated our analytical findings, showing that increasing the number of RIS elements and optimizing power allocation can substantially improve performance. Furthermore, the asymptotic analysis revealed that the system’s reliability depends heavily on the fading severity, highlighting the importance of proper system design. Future research could investigate the effects of imperfect CSI both in estimation and quantization on RIS phase-shift design, while also examining energy-efficiency versus reliability trade-offs under practical hardware and power-harvesting constraints. These studies will further guide the optimal deployment of RIS-enabled backscatter systems in 6G and IoT networks.

## Supporting information

S1_MATLAB_code (ZIP)
