# Supplementary figures and images for: Enhancing short-packet communications: BLER performance in RIS-assisted ambient backscatter NOMA systems
Source: PLoS One. 2025 Aug 5;20(8):e0328545. doi: 10.1371/journal.pone.0328545 (PMC12324139; doi:10.1371/journal.pone.0328545)

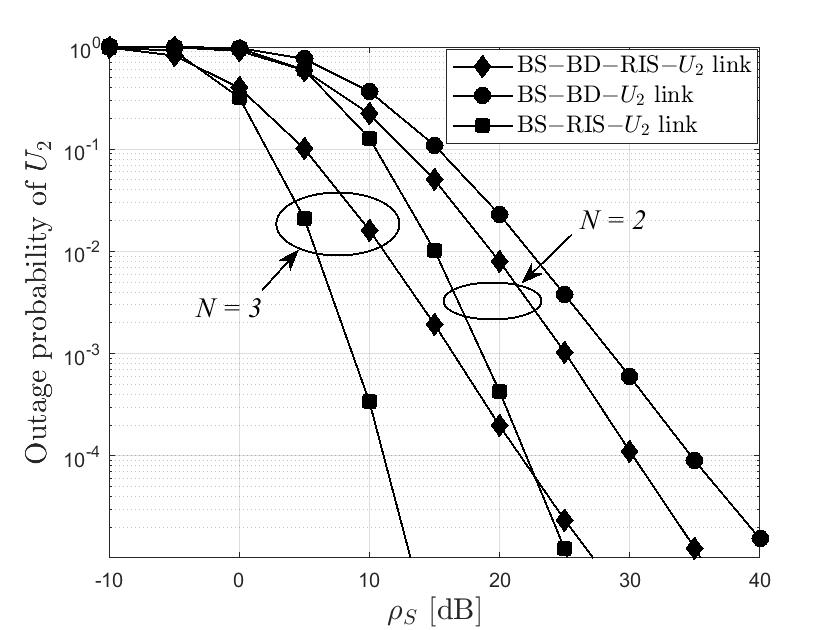

Supplement: S1_MATLAB_code — (ZIP) [file pone.0328545.s001.zip › code/SoSanh.jpg]
